# Supplementary material for: Predictors of self-reported practice in ventilator-associated pneumonia (VAP) prevention among critical care nurses in Sarawak public hospitals
Source: PLoS One. 2025 Dec 16;20(12):e0325637. doi: 10.1371/journal.pone.0325637 (PMC12707642; doi:10.1371/journal.pone.0325637)
Supplement: S1 Table — (DOCX) [file pone.0325637.s001.docx]

**Table S1: Item Analysis of Critical Care Nurses’ Knowledge Towards Ventilator-Associated Pneumonia (VAP) Prevention.**

| **Knowledge items** | **Correct (%)** | **Incorrect (%)** |
| --- | --- | --- |
| Regular comprehensive oral care (by tooth brushing and rinsing with antiseptic agent) | 245 (82.0) | 53 (18.0) |
| Frequency of ventilator circuit changes | 223 (75.0) | 75 (25.0) |
| Type of airway humidifier | 223 (75.0) | 74(25.0) |
| Oral versus nasal route for endotracheal intubation | 213 (71.0) | 85 (29.0) |
| Regular emptying of condensate from the ventilator tubing | 209 (70.0) | 89 (30.0) |
| Noninvasive mechanical ventilation (NIMV) | 153 (51.0) | 145 (49.0) |
| Daily sedation interruption and assessment of readiness for weaning | 152 (51.0) | 146 (49.0) |
| Patient positioning in bed | 132 (44.0) | 166 (56.0) |
| Open versus closed-circuit suction systems | 115 (39.0) | 183 (61.0) |
| Prevention of unplanned extubation and subsequent re-intubation | 94 (32.0) | 204 (68.0) |
| Frequency of closed-circuit suctioning system changes | 91 (31.0) | 207 (69.0) |
| Type of endotracheal tubes (ETTs) | 69 (23.0) | 229 (77.0) |
| Kinetic (mechanical) versus standard beds | 69 (23.0) | 229 (77.0) |
| Frequency of humidifiers (HME) changes | 5 (2.0) | 293 (98.0) |

*Note:* %: percentage
