# Supplementary material for: Predictors of self-reported practice in ventilator-associated pneumonia (VAP) prevention among critical care nurses in Sarawak public hospitals
Source: PLoS One. 2025 Dec 16;20(12):e0325637. doi: 10.1371/journal.pone.0325637 (PMC12707642; doi:10.1371/journal.pone.0325637)
Supplement: S2 Table — (DOCX) [file pone.0325637.s002.docx]

**Table S2: Item Analysis of Critical Care Nurses’ Self-Reported Practice Towards Ventilator-Associated Pneumonia (VAP) Prevention.**

| **Practice Items** | **Always (%)** | **Sometimes (%)** | **Never (%)** |
| --- | --- | --- | --- |
| Handwashing after any approach to a patient | 295 (99.0) | 2 (0.7) | 1 (0.3) |
| Handwashing before any approach to a patient | 294 (98.7) | 4 (1.3) | 0 |
| Use sterile gloves when open suction is necessary | 288 (96.6) | 9 (3.0) | 1 (0.3) |
| Use the heat and moisture exchanger humidifiers | 264 (88.6) | 30 (10.1) | 4 (1.3) |
| Change the closed-circuit suction systems for every new patient or when clinically indicated | 263 (88.3) | 34 (11.4) | 1 (0.3) |
| Change the heat and moisture exchanger humidifiers weekly (or when clinically indicated) | 250 (83.9) | 35 (11.7) | 13 (4.4) |
| Maintain the patient on semi fowler position | 248 (83.2) | 50 (16.8) | 0 |
| Provide regular oral care at least once per shift | 237 (79.5) | 61 (20.5) | 0 |
| Use of protective gloves at every approach to a patient | 235 (78.9) | 63 (21.1) | 0 |
| Interrupt sedation daily and assess readiness to extubate by daily spontaneous breathing trials | 227 (76.2) | 65 (21.8) | 6 (2.0) |
| Use of kinetic beds (special automatic beds that allow for frequent changing position) | 226 (75.8) | 51 (17.1) | 21 (7.0) |
| Provide scheduled and regular respiratory physiotherapy | 220 (73.8) | 71 (23.8) | 7 (2.3) |
| Change ventilator circuit only when visibly soiled or malfunctioning | 212 (71.1) | 67 (22.5) | 19 (6.4) |
| Use chlorhexidine solution for oral care | 181 (60.7) | 73 (24.5) | 44 (14.8) |
| Suction the subglottic secretions through an extra lumen in the endotracheal tubes when patients likely to require > 48 or 72 hours of intubation | 150 (50.3) | 103 (34.6) | 45 (15.1) |
| Use of close-circuit suction systems | 142 (47.7) | 156 (52.3) | 0 |

*Note:* %: percentage
