# Supplementary material for: Predictors of self-reported practice in ventilator-associated pneumonia (VAP) prevention among critical care nurses in Sarawak public hospitals
Source: PLoS One. 2025 Dec 16;20(12):e0325637. doi: 10.1371/journal.pone.0325637 (PMC12707642; doi:10.1371/journal.pone.0325637)
Supplement: S3 Table — (DOCX) [file pone.0325637.s003.docx]

**Table S3: Item Analysis of Critical Care Nurses’ Barriers Towards Ventilator-Associated Pneumonia (VAP) Prevention.**

| **Barriers Items** | **Agree (%)** | **Neither agree, nor disagree (%)** | **Disagree (%)** |
| --- | --- | --- | --- |
| There is a staff shortage in my unit | 227 (76.2) | 67 (22.5) | 4 (1.3) |
| Forgetfulness to perform some evidence-based procedure may increase the risk of VAP | 84 (28.2) | 162 (54.4) | 52 (17.4) |
| There is no written protocol on ventilator associated pneumonia management | 53 (17.8) | 96 (32.2) | 149 (50.0) |
| Cost control policies in my hospital negatively affect patient’s quality of care | 52 (17.4) | 153 (51.3) | 93 (31.2) |
| There are no educational courses on ventilator-associated pneumonia management | 35 (11.7) | 163 (54.7) | 100 (33.6) |
| There are no enough resources and supplies example sterile gloves, closed system suction, kinetic beds, & others | 31 (10.4) | 102 (34.2) | 165 (55.4) |
| There was no proper education study about ventilator associated pneumonia management/prevention during my basic training | 26 (8.7) | 131 (44.0) | 141 (47.3) |
| The current practices in my unit to decrease ventilator- associated pneumonia (VAP) are not based on research evidence | 23 (7.7) | 109 (36.6) | 165 (55.7) |
| There was insufficient training in my unit to guide me for appropriate VAP management | 23 (7.7) | 151 (50.7) | 124 (41.6) |
| Patients don’t cooperate when I perform some procedures to decrease VAP | 14 (4.7) | 235 (78.9) | 49 (16.4) |
| Some procedures related to VAP management are out of my job description | 13 (4) | 134 (45.0) | 151 (50.7) |
| I don’t find enough time to perform some procedures in the right way to decrease VAP | 10 (3.4) | 144 (48.3) | 144 (48.3) |

*Note:* %: percentage
