# Supplementary material for: Predictors of self-reported practice in ventilator-associated pneumonia (VAP) prevention among critical care nurses in Sarawak public hospitals
Source: PLoS One. 2025 Dec 16;20(12):e0325637. doi: 10.1371/journal.pone.0325637 (PMC12707642; doi:10.1371/journal.pone.0325637)
Supplement: Attachment — Submitted filename: Response to reviewers.docx [file pone.0325637.s005.docx]

Dear editor,

Thank you very much for your professionalism and consideration on our manuscript. The reviewers’ constructive comments and suggestions have greatly help us improved the quality of the manuscript. We have done major revision based on the comments. We answer all of the questions or comments in details in the following summary table.

|  | Topic / subtopic | Comments from reviewers | Action taken |
| --- | --- | --- | --- |
| REPLY TO 1^st^ REVIEWER | | | |
|  | Topic / subtopic | Reviewer’s comments | Action taken |
| 1 | Terminology / Clarity of Measures | - The term "perceived practice" is used throughout the manuscript but is conceptually vague. Instead self reported practice may be used - Self report introduces bias (social desirability bias) which is not described in the discussion part. This may be responsible for the high compliance reported despite poor knowledge. - Variables such as workplace culture, availability of clinical protocols, and team dynamics are not explored but likely influence practice, can be included in the discussion of limitations | We thank the reviewer for these constructive comments, which have helped to improve the clarity and rigor of the manuscript. In response:   - The term “perceived practice” has been replaced with “self-reported practice” consistently throughout the manuscript (Methods, Results, and Discussion sections) to ensure clarity. - We acknowledge that reliance on self-reported practice may introduce social desirability bias, which could partly explain the high compliance rates despite low knowledge. This has now been addressed in the discussion and limitations section. - In addition, we have added a statement to the limitations noting that workplace culture, clinical protocols, and team dynamics were not examined in this study, even though these factors are likely to influence practice (page X, lines 433–435). |
| 2 | References | Minor concerns- YouTube link may not be appropriate as reference   - Faculty of Medicine University Malaya. Antimicrobial Resistance: Combating the silent 443 pandemic mini-symposium [Internet]. YouTube; 2022 Sep 10 [cited 2024 Apr 10]. 444 Available from: https://www.youtube.com/watch?v=HXF9mh6O2TQ. —- YOUTUBE LINK AS REFERENCE) | - The YouTube reference has been removed from the reference list, and the related statement in the manuscript has also been deleted or replaced with a peer-reviewed source (lines 59–60). |
| REPLY TO 2^nd^ REVIEWER | | | |
|  | Topic / subtopic | Reviewer’s comments | Action taken |
| 1. | Methods  - Data collection  -Measurement | Authors mentioned “Responses with incomplete data or entry errors were excluded.” What was the threshold used? Any data missing or specific/critical data? If so, what are the critical data that lead to exclusion. | - We appreciate the reviewer’s request for clarification. We have now specified how incomplete data were defined and the number of questionnaires excluded. - The methods section has been revised to state that incomplete data were defined as any questionnaire with missing responses to demographic, knowledge, or practice items, since all items were considered essential for analysis. Only fully completed questionnaires were retained. A total of 12 incomplete questionnaires were excluded, leaving 298 valid responses for analysis (lines 121-125). |
| 4. |  | Knowledge of VAP prevention strategies The total scale had a Cronbach's α of .64, the CVI was .89, and this scale was valid and reliable. Please spell out CVI. | - We thank the reviewer for pointing this out. - CVI has been spelled out in full as Content Validity Index in the Methods section (line 147-148). |
| 3. | Results | What was the response to questionnaire? How many nurses were invited to participate and how many responded? Response rate is important for the point of view of reproducibility and reliability of results. | - We appreciate the reviewer’s observation and agree that reporting the response rate is important for reproducibility and reliability. - We have added details to the Methods/Results section stating that out of 454 eligible critical care nurses, the minimum required sample size was 213 (calculated using Yamane’s formula). After adjusting for a 20% attrition rate, the final target sample size was 267. A total of 298 nurses completed the questionnaire, representing a response rate of 65.6%, which exceeded the required sample size and supports the reliability and generalizability of the findings (lines 194-198). |
|  |  | Line 192-193 - Most participants were clinical staff nurses (74.8%) with 58.1% working fewer than 45 hours per week, and 98.0% were permanent staff members. The statement is confusing. I assume that all participants in this study were nurses. Then, what is meaning of “Most participants were clinical staff nurses (74.8%)”? Please clarify or rephrase the sentence. | - We thank the reviewer for this observation and agree that clarification was needed. - The sentence has been revised to state that all participants were nurses. The majority (74.8%) were bedside clinical staff nurses (entry-level registered nurses), while 20.5% were senior registered nurses with 13–15 years of service, promoted through structured time-based pathways, who frequently take on supervisory and mentoring roles in addition to their clinical duties. A smaller proportion (4.7%) were nurse managers (ward or unit leaders with broader administrative responsibilities). (Lines 206-210) |
|  |  | Table 1. What is “Post basic ICU’? Please use a terminology which is self-explanatory. Also, use full name for Pead, Neuro, ID, SN… | - We appreciate the reviewer’s comment and agree that clearer terminology should be used. - The term “Post basic ICU” has been replaced with “advanced specialty training in intensive care nursing (locally termed post-basic qualification in ICU)” for clarity. In addition, all abbreviations (Pead, Neuro, ID, SN) have been replaced with their full forms in Table 1 (page 10-11). |
|  |  | Table 2. Table has classified participants in to Good, Acceptable and Poor Knowledge. However, the basis of classification is not clear from the manuscript. | - We thank the reviewer for pointing this out and agree that the classification method should be clearly described. - The methods section has been revised to specify that categorized according to Al-Sayaghi’s cut-off points: Good (70–100%), Acceptable (60–69%), and Poor (≤60%) (Lines 145-147). |
|  |  | Table 3  The title and variable for practice levels are confusing. It is not clear from the text what exactly is this table depicting. Does it depict – Perceived compliance to VAP preventive strategies Or is it actual compliance to the VAP Preventive strategies? Also, in table variable compliance and knowledge both are mentioned, which variable is correct? | - We appreciate the reviewer’s observation and agree that clarification was needed regarding Table 3. - The text and table have been revised to specify that the data represent self-reported practices (perceived compliance) towards VAP prevention. In line with Al-Sayaghi’s classification, practice levels are now grouped into three categories: High compliance, Acceptable compliance, and Unsafe practice. References to “knowledge” in this table have been corrected to avoid confusion (lines 156-158; Table 3). |
|  |  | Table 4 It is not clear. The table does not present any relationship/correlation (e.g. co-relation coefficient). Please modify the table. Also, table is usually used to present multiple variables/outcomes together for easy understanding. However, in this table only 1 variables are there. So, please think of alternative way of presentation. | - We thank the reviewer for this valuable suggestion. - The table has been revised and the title updated to: “Table 4. Correlation between knowledge and self-reported practice of ventilator-associated pneumonia (VAP) prevention among critical care nurses.” - The two variables are now labeled (1 = Knowledge, 2 = Practice) and presented side by side in a correlation matrix with the correlation coefficient included. - The data analysis section was also updated to specify that “Pearson’s correlation analysis was used to examine the relationship between knowledge and self-reported practice towards VAP prevention.” (lines 270-271; Table 4). |
|  |  | Predictors of critical care nurses’ practice in VAP prevention. Authors have described model 1 and model 2. However, the basis of modelling is not described in the methodology. | - We thank the reviewer for this important comment and agree that the modelling approach needed clarification. - The data analysis section has been revised to explain the basis of the hierarchical multiple regression modelling. Specifically, Model 1 included organizational and workload-related variables (type of unit, number of official beds, and nurse–patient ratio for ventilated patients), while Model 2 added knowledge score to test its additional contribution beyond these factors. Statistical significance was set at p < .05 (lines 186-190). |
|  | References | References should be preferable from Journals. However, there are many citations from the internet source. For example – ref 22. There are many trials on this subject. May cite the latest systematic review/metanalysis. Other references - 31 | We appreciate this observation.   - We agree that journal references are preferable and have ensured that most of our references are from peer-reviewed journals. For a few sources, no DOI was available, so we cited them as [Internet] with the date of access and the official link, following Vancouver style. These were included only where they provide essential context, such as local nursing studies, Ministry of Health policies, and CDC guidelines, which are not available elsewhere in indexed journals. We have also reviewed our reference list and retained only the most relevant and credible internet sources. - References 10,11,13,15,22,28,30,31, and 40   We also appreciate the reviewer’s suggestion and agreeing that peer-reviewed journal references are more appropriate.   - References 22 and 31 (previously non-journal sources) have been removed and replaced with recent systematic reviews/meta-analyses: - Ref 22: Pozuelo-Carrascosa DP, Herráiz-Adillo Á, Alvarez-Bueno C, Añón JM, Martínez-Vizcaíno V, Cavero-Redondo I. *Subglottic secretion drainage for preventing ventilator-associated pneumonia: An overview of systematic reviews.* Eur Respir Rev. 2020;29(155):190107. doi:10.1183/16000617.0107-2019. - Ref 31: De Cassai A, Pettenuzzo T, Busetto V, Legnaro C, Pretto C, Rotondi A, Boscolo A, Sella N, Munari M, Navalesi P; et al. Chlorhexidine is not effective at any concentration in preventing ventilator-associated pneumonia: A systematic review and network meta-analysis. J Anesth Analg Crit Care. 2024;4(1):30. doi:10.1186/s44158-024-00166-2.   (Page 25-26, reference list). |
