# Supplementary material for: Predictors of self-reported practice in ventilator-associated pneumonia (VAP) prevention among critical care nurses in Sarawak public hospitals
Source: PLoS One. 2025 Dec 16;20(12):e0325637. doi: 10.1371/journal.pone.0325637 (PMC12707642; doi:10.1371/journal.pone.0325637)
Supplement: Attachment — Submitted filename: Response_to_reviewers_auresp_2.docx [file pone.0325637.s006.docx]

Dear Editor,
Thank you very much for your continued consideration of our manuscript. We sincerely appreciate the reviewer’s constructive feedback, which has helped us further improve the clarity and presentation of our work.

As this round involved a minor revision, we have addressed the single comment regarding the combination of Table 3 and Table 4. These have now been merged into one table titled “Table 3. Critical care nurses’ self-reported practice levels and correlation with knowledge on ventilator-associated pneumonia (VAP) prevention (N=298)”

In the Results section, minor adjustments to paragraph order were made to accommodate the placement of the newly combined Table 3, no textual content was altered.

The corresponding text references have also been updated accordingly.

|  | Topic / subtopic | Comments from reviewers | Action taken |
| --- | --- | --- | --- |
| REPLY TO 2^nd^ REVIEWER | | | |
|  | Topic / subtopic | Reviewer’s comments | Action taken |
| 3. | Results | Please combine table 3 and 4. It will be better to presentation of the data in single table. | - Tables 3 and 4 have been merged into a single, integrated table titled “Table 3. Critical care nurses’ self-reported practice levels and correlation with knowledge on ventilator-associated pneumonia (VAP) prevention (N=298)” - Corresponding in-text references were updated accordingly. - This revision improves data presentation, with no changes to the results or interpretations. |
